# Combination of RNAseq and SNP nanofluidic array reveals the center of genetic diversity of cacao pathogen *Moniliophthora roreri* in the upper Magdalena Valley of Colombia and its clonality

**DOI:** 10.3389/fmicb.2015.00850

**Published:** 2015-08-27

**Authors:** Shahin S. Ali, Jonathan Shao, Mary D. Strem, Wilberth Phillips-Mora, Dapeng Zhang, Lyndel W. Meinhardt, Bryan A. Bailey

**Affiliations:** ^1^Sustainable Perennial Crops Laboratory, Plant Sciences Institute, United States Department of Agriculture/Agricultural Research Service, Beltsville Agricultural Research Center-WestBeltsville, MD, USA; ^2^Departamento de Agricultura y Agroforestería, Centro Agronómico Tropica de Investigación y EnseñanzaTurrialba, Costa Rica

**Keywords:** RNAseq, SNP, monilia pod rot, genotyping, biodiversity, homothallism

## Abstract

*Moniliophthora roreri* is the fungal pathogen that causes frosty pod rot (FPR) disease of *Theobroma cacao* L., the source of chocolate. FPR occurs in most of the cacao producing countries in the Western Hemisphere, causing yield losses up to 80%. Genetic diversity within the FPR pathogen population may allow the population to adapt to changing environmental conditions and adapt to enhanced resistance in the host plant. The present study developed single nucleotide polymorphism (SNP) markers from RNASeq results for 13 *M. roreri* isolates and validated the markers for their ability to reveal genetic diversity in an international *M. roreri* collection. The SNP resources reported herein represent the first study of RNA sequencing (RNASeq)-derived SNP validation in *M. roreri* and demonstrates the utility of RNASeq as an approach for *de novo* SNP identification in *M. roreri*. A total of 88 polymorphic SNPs were used to evaluate the genetic diversity of 172 *M. roreri* cacao isolates resulting in 37 distinct genotypes (including 14 synonymous groups). Absence of heterozygosity for the 88 SNP markers indicates reproduction in *M. roreri* is clonal and likely due to a homothallic life style. The upper Magdalena Valley of Colombia showed the highest levels of genetic diversity with 20 distinct genotypes of which 13 were limited to this region, and indicates this region as the possible center of origin for *M. roreri*.

## Introduction

*Moniliophthora roreri* H. C. Evans, Stalpers, Samson, and Benny is the causal agent of frosty pod rot (FPR) of *Theobroma cacao* L. (Evans et al., 1978), a major cash crop in the tropics and the source of chocolate. FPR occurs in most major cacao producing countries in the Western Hemisphere, other than Brazil (Phillips-Mora et al., 2007a), causing yield losses up to 80% (Hidalgo et al., 2003). *M. roreri* is a hemibiotrophic fungus. During the biotrophic phase the fungus slowly colonizes the fruit causing malformations and positions itself to exploit the nutritive resources released when necrosis is triggered. A major shift in *M. roreri* gene expression occurs between the biotrophic and necrotrophic phases, and pod metabolites are rapidly metabolized by the pathogen during the necrotrophic phase and finally sporulation occurs (Bailey et al., 2013). Dikaryotization of the fungal mycelia from a haploid state is thought to occur in association with the shift to the necrotrophic phase.

As chemical control of FPR is not generally economically feasible (Bateman et al., 2005), an integrated disease management approach involving better agronomic practices combined with improved planting materials and possibly biological control approaches (Krauss and Soberanis, 2001; Krauss et al., 2010; de la Cruz et al., 2011), is the only feasible strategy for managing FPR at this moment. Though there are no cacao clones immune to *M. roreri*, highly tolerant clones have been developed by CATIE in Costa Rica (Phillips-Mora et al., 2005; Phillips-Mora, 2010). In addition, cacao clones like EET 233 and ICS 95 have demonstrated tolerance against FPR in screening studies conducted in Ecuador and Peru (Evans, 1981; Evans et al., 1998). Tolerance against *M. roreri* includes the induction of cacao defense responsive genes early in the infection process compared to that observed in susceptible clones (Ali et al., 2014). Losses to FPR in most CATIE selected tolerant clones has slowly increased over the past 15 years (Phillips-Mora et al., 2012) raising questions that the pathogen may be in the process of overcoming or “breaking” the tolerance. The pathogen has the potential to employ an intricate pattern of alternative gene expression when sucessfully causing disease in tolerant cacao clones (Bailey et al., 2014).

Though Ecuador was traditionally considered as the origin of FPR, recent reports have shown a higher genetic diversity in Colombia, which would make Colombia the true center of origin of *M. roreri* on cacao (Phillips-Mora et al., 2007a). Until the 1970s, FPR was confined to Colombia, Ecuador, western Venezuela and eastern Panama (Phillips-Mora et al., 2007b) and was regarded as a geographically isolated pathogen (Holliday, 1971). It then moved northward into Central America through Panama and reached Costa Rica in the late 1970s (Enríquez and Suarez, 1978), where production fell fivefold due to FPR (Hidalgo et al., 2003). FPR has continued to spread northward, with its presence being confirmed in Guatemala, Honduras, and Mexico (Phillips-Mora et al., 2006, 2007a,b). The spread of the disease into Central America may have been by airborne spores; however, movement as a result of human activities is considered more likely (Orellana, 1956). On the other hand, the natural dispersion of the FPR disease from western Ecuador to areas further south and east was limited by the Andes, an impassable barrier that limited the spread of the disease until the 1980s (Evans, 1986). The introduction of the FPR disease into the Amazonian regions of Eastern Ecuador coincided with new agricultural development, the construction of oil pipelines and roads into the Eastern Ecuador. From there, the pathogen dispersed southward along the eastern slopes of the Andes reaching first Peru (Hernández et al., 1990) and, more recently, Bolivia (Phillips-Mora et al., 2012).

Genetic diversity within the population of a fungal pathogen allows specific individuals in the population to adapt to changing environmental conditions, particularly to enhanced resistance or tolerance of the host, which is developed by natural selective pressures or introduced by plant breeders (Wang and Szmidt, 2000; Carlile et al., 2001). Therefore a proper understanding of the genetic variation in this plant pathogen is critical to the establishment of an effective and sustainable resistance breeding program. Amplified fragment length polymorphisms (AFLP) and inter simple sequence repeat (ISSR) marker analysis showed that *M. roreri* has five genetic groups (Phillips-Mora et al., 2007a), the two major ones being the Bolivar group, comprising isolates from Colombia, Peru, Venezuelan, and Ecuador and the Co-West group, comprising isolates from Colombia, Ecuador, and Central America. The other three groups are endemic to Colombia and North-Western Ecuador. The recent designation of North-Western Colombia as the center of diversity and origin of FPR (Phillips-Mora et al., 2007a) makes it especially important to examine, the genetic diversity of the pathogen in this region in depth. This knowledge is necessary when establishing and maintaining breeding programs for disease tolerance and in developing and deploying integrated disease management strategies that include the planting of tolerant cacao clones.

Although ISSR and AFLP markers are useful in assessing genetic diversity, it is important for long term studies to develop co-dominant molecular markers that can accurately identify each individual isolate and give more consistent quantitative results. Single nucleotide polymorphisms (SNPs) represent the most copious source of genetic variation and are linked to heritable differences between individual strains of a pathogen (Suh and Vijg, 2005). Since the *M. roreri* genome was recently sequenced using an Ecuadorian isolate (Meinhardt et al., 2014), discovering SNPs by comparison of NGS data with the genomes or ESTs could be an effective tool. Exome sequencing (Ng et al., 2009) is based on the hypothetical foundation that the majority of pathogenicity related mutations are located within coding sequences. Though RNA sequencing (RNASeq) is mainly considered a method of gene expression analysis, it is also a form of exome sequencing with the capacity to detect SNPs in the genes that are expressed (Quinn et al., 2013). SNP detection using RNASeq results has been validated in many studies (Chepelev et al., 2009; Cánovas et al., 2010; Salem et al., 2012; Vidal et al., 2012).

The objectives of the present study were to develop SNP markers through the data mining and comparison of RNASeq results from 13 *M. roreri* isolates and the reference genome sequence (Meinhardt et al., 2014) and access these SNP markers for their ability to reveal genetic diversity in an international *M. roreri* culture collection. The SNP resources reported herein represent the first study of RNASeq based SNP discovery and validation in *M. roreri*, demonstrating the utility of RNASeq results as an approach for *de novo* SNP identification in this pathogen. These SNP markers, as well as the genotyping method developed, will allow researchers to address key questions about the population genetics of *M. roreri*. For example SNP analysis could help us understand: recombination events, fungal mating types, pathogen genetics, and population adaptations resulting from changes in the host genetics, and help predict the stability of new tolerant host plants developed by plant breeding programs. In this study, we identify and characterized 88 SNP markers in *M. roreri* and use them to assess the genetic diversity of *M. roreri* across its geographic range.

## Materials and Methods

### *Moniliophthora roreri* Isolates

Most isolates used in this study belong to CATIE’s collection of *M. roreri* collected from affected areas of South and Central America from 1999 to 2013 (See Supplementary Excel file S1). All samples were obtained from infected fruits of *T. cacao*. A significant percentage of all isolates were collected from Colombia, since Colombia is now presumed to be the center of origin of the pathogen (Phillips-Mora et al., 2007a). A considerable representation of isolates were also obtained from Costa Rica since that is where the CATIE breeding program resides and to compare changes in genetic diversity of the fungus post dispersal from the presumed center of origin. Two additional *M. roreri* isolates were obtained from the ATCC(ATCC42952 and ATCC64239) that were isolated in 1978 and 1984, respectively, from Costa Rica. *M. roreri* cultures were isolated from pods showing the initial or intermediate stages of external necrosis. Pods were cut with a sterile knife and small internal tissue sections from the zone where necrotic and healthy tissues meet were removed to water agar plates. Cultures typical of *M. roreri*, slow growing white mycelia, were transferred to V8 medium (20% w/v V8 juice, 0⋅1% w/v asparagine, 2⋅0% w/v maltose, 1⋅8% w/v agar). Isolates were shipped to USDA-APHIS-PPQ facility in Beltsville, MD and transferred to USDA-ARS Sustainable Perennial Crops Lab in Beltsville, MD after inspection.

### DNA and RNA Extraction

For DNA extraction, 2–3 agar plugs (0.25 cm^2^) were transferred from cultures grown on V8 media to 50 ml falcon tubes containing 20 ml liquid clarified V8 media (Drenth and Sendall, 2001) and grown at room temperature while shaking at 100 rpm. Cultures were allowed to grow for 15–20 days. Mycelia were washed with sterile water and collected by centrifuging at 20,000 *g* for 10 min in the same falcon tube followed by flash freezing in liquid nitrogen and freeze drying. Genomic DNA was extracted as mentioned by Ali et al. (2014).

For RNA extraction, 2–3 agar plugs were transferred from cultures grown on V8 agar plate to 250 ml conical flask containing 50 ml potato dextrose broth and grown at room temperature (22–25°C) while shaking at 100 rpm. Cultures were allowed to grow for 10–15 days. Mycelia were washed with sterile water and collected by centrifuging at 20,000 *g* for 10 min followed by flash freezing in liquid nitrogen and freeze drying. Freeze-dried mycelia were ground in a mortar and pestle in liquid nitrogen and transferred to a 50 mL centrifuge tube containing 15 mL of 65°C extraction buffer (Bailey et al., 2005). Additional extraction methods were conducted as in Bailey et al. (2013). Using a NanoDrop spectrophotometer (Thermo Scientific, Wilmington, DE, USA), RNA and DNA concentrations were determined by absorbance at 260 nm and purity was estimated by the 260/280 ratio and the 260/230 ratio.

### RNASeq Analysis and Mining of Putative SNPs

**FIGURE 1:**
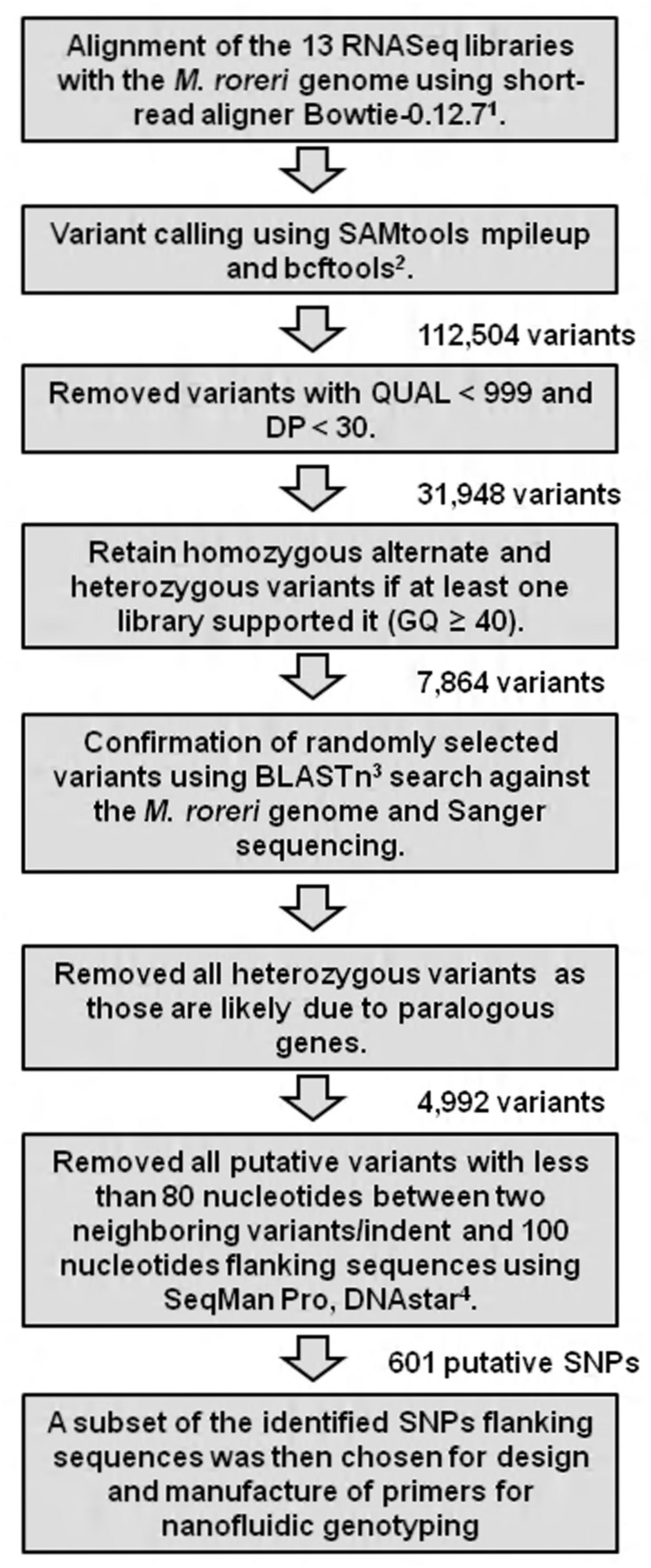
**The overview of the single nucleotide polymorphism (SNP) mining strategy from *Moniliophthora roreri* RNASequencing (RNASeq) libraries.** Each library from *M. roreri* isolates Co17, Co7, E18, Co15, E16, E43, C13, Co12, Co8, Co11, E32, P1, and MCA2977 was aligned against the *M. roreri* genome (Meinhardt et al., 2014) and variant calling was performed. Reference for bioinformatics tools: ^1^Langmead et al., 2009;^2^Li et al., 2009; ^3^Larkin et al., 2007, and ^4^DNASTAR, 1999. Abbreviation: QUAL (quality): Phred-scaled quality score for the assertion made in alternate base; DP: Combined depth across samples; GQ: Conditional genotype quality.

RNA Sequencing analysis was carried out by the National Center for Genome Resources (Santa Fe, NM, USA). For the RNASeq analysis, cDNA was generated using a routine RNA library preparation TruSeq protocol developed by Illumina Technologies (San Diego, CA, USA). Using the kit, mRNA was first isolated from total RNA by performing polyA selection step, followed by construction of single end sequencing libraries with an insert size of 160 bp. Single-end sequencing was performed on 13 samples using the Illumina HiSeq platform. Samples were multiplexed with unique six-mer barcodes generating 627,844,665 filtered (for Illumina adapters/primers, and PhiX contamination) 1 × 50 bp read pairs. Each library (Co17, Co7, E18, Co15, E16, E43, C13, Co12, Co8, Co11, E32, P1, and MCA2977) ranging from 20 to 62 million reads in fastq format was aligned to the *M. roreri* (MCA2977) genome sequences (Meinhardt et al., 2014) using a memory-efficient short-read aligner Bowtie-0.12.7 (Langmead et al., 2009). The files were outputted into SAM (Sequence Alignment/Map) format. The files were converted to BAM format, a binary format for storing sequence data and sorted using SAMtools (Li et al., 2009). Variant calling was performed using SAMtools mpileup and bcftools with default parameters, which generated a VCF file (variant call format; Li et al., 2009). The resulting VCF file was filtered manually using Excel functions. All SNPs with QUAL < 999 and DP < 30 were removed (Danecek et al., 2011). SNPs were further filtered based on genotype quality (GQ) and a homozygous alternate (1/1) or heterozygous (0/1) SNP call was retained if there was at least one library supporting it (GQ ≥ 40; See Supplementary Excel file S2). SNPs were further verified and manually visualized using the bioinformatics suite DNASTAR using the program SeqMan Pro (DNASTAR, 1999). In order to meet the requirements and constraints for primer design, all candidates for SNP markers with less than 80 nucleotides between two neighboring SNPs, and with flanking sequences less than 100 nucleotides long, were removed. The overview of the SNP mining strategy has been presented in **Figure 1**. A subset of the identified SNP flanking sequences was then chosen for design and synthesis of primers to assay for SNPs (See Supplementary Excel file S2).

### Confirmation of *In Silico* SNPs

To confirm that the *in silico* generated SNPs are not due to paralogous genes, we randomly selected 25 putative homozygous and heterozygous SNPs and performed BLASTn searches of the ∼100 bp flaking regions against the *M. roreri* MCA 2997 (taxid:221103) whole-genome shotgun contigs. As the homozygous SNPs showed single hits while the heterozygous SNPs showed multiple hits within the *M. roreri* genome (See Supplementary Excel file S3), one homozygous and four heterozygous SNPs were further confirmed by Sanger sequencing. The PCR-amplified products flanking putative homozygous and heterozygous SNP markers from different *M. roreri* isolates were recovered from agarose gels and purified using QIAGEN gel purification kits (QIAGEN, Dusseldorf, Germany; See Supplementary Table S1 for primers used for PCR amplifications). The purified amplicons were cloned into pCR4-TOPO vector (TOPO TA Cloning Kit, Life Technologies, USA) and transformed in to DH5-alpha *Escherichia Coli* (New England Biolab, USA) according to the manufacturers protocol. Then 9–12 clones from each amplicon were randomly selected to be commercially sequenced (Macrogen, Rockville, MD, USA) from both ends using M13F and M13R primers. After removing the vector sequences, ClustalW2 (Larkin et al., 2007) was used to compare the DNA sequences of each clones and the original sequences containing the SNP.

### Design of the SNP Panel and Validation of Putative SNPs

To evaluate the putative SNP markers for suitability of genotyping the *M. roreri* isolates, a nanofluidic genotyping system was used to validate the SNPs for 172 samples (See Supplementary Excel file S1). A total of 288 SNP sequences were submitted to the Assay Design Group at Fluidigm Corporation (South San Francisco, CA, USA) for design and manufacture of primers for a SNPtype^TM^ genotyping panel. The assays were based on competitive allele-specific PCR and enable bi-allelic scoring of SNPs at specific loci (KBioscience Ltd., Hoddesdon, UK). The Fluidigm SNPtype^TM^ Genotyping Reagent Kit was used according to the manufacturer’s instructions. Using these primers, the isolated DNAs were subjected to Specific Target Amplification (STA) in order to enrich the SNP sequences of interest. Genotyping was performed on a nanofluidic 96.96 Dynamic Array IFC (Integrated Fluidic Circuit; Fluidigm Corp., South San Francisco, CA, USA). This chip automatically assembles PCR reactions, enabling simultaneous testing of up to 94 samples with 96 SNP markers. The use of a 96.96 Dynamic Array^TM^ IFC for SNP genotyping of human samples was as described by Wang et al. (2009). End-point fluorescent images of the 96.96 IFC were captured and processed with an EP1^TM^ imager (Fluidigm Corp., CA, USA). The data was analyzed with Fluidigm Genotyping Analysis Software (Fluidigm SNP genotyping user guide, Fluidigm, South San Francisco, CA, USA).

### Validation of Heterozygous SNP Calls by the Fluidigm System

To validate the heterozygous SNP calls by the Fluidigm system, two such putative SNPs were tested for three *M. roreri* isolates using Sanger sequencing. The PCR-amplified products flanking putative SNP markers from different *M. roreri* isolates were recovered from agarose gels and purified using QIAGEN gel purification kits (QIAGEN, Dusseldorf, Germany; See Supplementary Table S1 for primers used for PCR amplifications). The purified amplicons were cloned into pCR4-TOPO vector (TOPO TA Cloning Kit, Life Technologies, USA) and transformed in to DH5-alpha *E. Coli* (New England Biolab, USA) according to the manufacturers protocol. Then 9–12 clones from each amplicon were randomly selected to be commercially sequenced (Macrogen, Rockville, MD, USA) from both ends using M13F and M13R primers. After removing the vector sequences, ClustalW2 (Larkin et al., 2007) was used to compare the DNA sequences of each clones and the original sequences containing the SNP.

### Data Analysis

For genotype identification, pair-wise multilocus matching was applied among individual samples using the program GenAlEx 6.5 (Peakall and Smouse, 2006, 2012). DNA samples that were fully matched at the genotyped SNP loci were declared the same genotype (or clones). Distance-based multivariate analysis was used to assess the relationship among the individual isolates. Pair-wise genetic distances as defined by Peakall et al. (1995) were computed using the DISTANCE procedure implemented in GenAlEx 6.5. The same program was then used to perform Principal Coordinates Analysis (PCoA), based on the pair-wise distance matrix. Both distance and covariance were standardized. Cluster analysis was used to further examine the genetic relationship among isolates. Nei et al.’s (1983) genetic distance was calculated using Microsatellite Analyzer (MSA; Dieringer and Schlötterer, 2003) and 100 bootstrap replications were applied. A consensus dendrogram was generated from the resulting distance matrix using the neighbor-joinig algorithm (Saitou and Nei, 1987) available in PHYLIP (Plotree and Plotgram, 1989), and visualized using Figtree ver. 1.3.1 (Rambaut, 2009).

## Results

### RNASeq Analysis and SNP Discovery

The number of quality RNA reads (>50 bp) from RNASeq libraries ranged from 20 to 62 million reads (**Table 1**). After aligning the reads against the *M. roreri* reference genome sequence (Meinhardt et al., 2014) variant calling generated 112,504 putative SNPs in 2,350 *M. roreri* reference genome contigs. After filtering the SNPs as mentioned in the methods, a total of 7,862 putative CDS (coding DNA sequence)-based SNPs were obtained (See Supplementary Excel file S2). There were 6,329 transition type SNPs, including 3,191 C/T and 3,138 A/G; while there were 1,497 transversion type SNPs, including 283 A/T, 427 A/C, 390 T/G, 397 C/G, and 36 tri-allelic polymorphisms. Among the 12 *M. roreri* libraries, the total number of putative SNPs (QUAL ≥ 999, DP ≥ 30, and GQ ≥ 40) ranged between 224 and 2,340 (**Table 1**). The ratios between homozygous and heterozygous SNPs ranged between 1.9 and 0.1 (**Table 1**). Among the 25 randomly selected homozygous and heterozygous SNPs tested, none of the homozygous SNPs containing flanking regions showed more than one hit (*E* > 2*e*–50) within the *M. roreri* genome while all but one heterozygous SNP containing flanking regions showed two or more hits (*E* > 2*e*–50) with the exception being SNP 485_1_1286 (See Supplementary Excel file S3). This obviously raised a question concerning the validity of heterozygous SNP calls necessitating the validation of heterozygous SNPs by Sanger sequencing.

**Table 1:** List of RNASequencing (RNASeq) libraries.

*Moniliophthora roreri* isolates	Origin	Genetic group^#^	No. of RNA reads in each library	No. of single nucleotide polymorphisms (QUAL ≥ 999, DP ≥ 30, and GQ ≥ 40)	
				Homozygous	Heterozygous
C13	Costa Rica	IV, Co-West	51,121,595	948	807
Co12	Colombia	II, Co-Central	20,354,728	1539	801
Co8	Colombia	IV, Co-West	51,581,527	348	381
Co15	Colombia	III, Co-East	50,369,121	463	516
Co11	Colombia	II, Co-Central	50,921,967	283	477
Co17	Colombia	II, Co-Central	49,462,215	258	371
Co7	Colombia	II, Co-Central	49,467,859	458	276
E16	Ecuador	IV, Co-West	52,294,090	377	548
E18	Ecuador	IV, Co-West	44,491,879	262	428
E32	Ecuador	V, Boliver	62,268,785	436	578
E43	Ecuador	I, Gileri	52,827,877	804	531
P1	Peru	V, Boliver	44,820,900	24	218
MCA2977	Ecuador	Not known	47,862,122	41	306

*^#^As described by Phillips-Mora et al. (2007a).*

**FIGURE 2:**
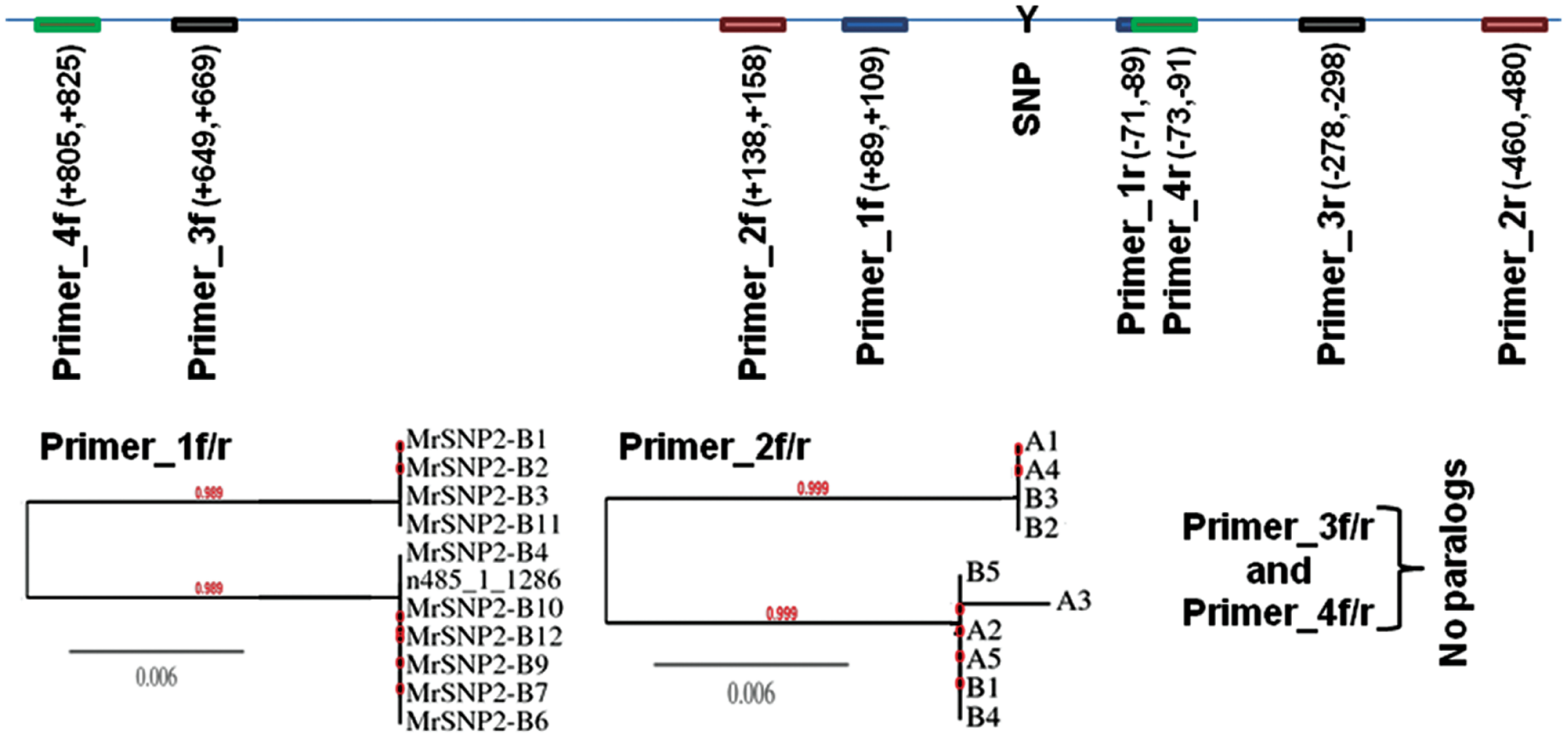
**Sanger sequencing of partial genomic DNA flanking the putative heterozygous SNP marker 485_1_1286 as called by SAMtools mpileup and bcftools with default parameters from *M. roreri* isolates C13.** PCR-amplicons were cloned into pCR4-TOPO vector followed by transformation into DH5-alpha *Escherichia coli* and 9–11 clones from each amplicon were randomly selected to be sequenced from both ends using M13F and M13R primers. ClustalW2 (Larkin et al., 2007) was used to compare the DNA sequences of each clone and a distance tree of 100 bootstrapped data sets was generated by using the Phylogeny.fr program (http://phylogeny.lirmm.fr/) and the neighbor-joining method.

Sanger sequencing of PCR-amplicons containing a putative homozygous SNP from three *M. roreri* isolates validated the presence of that homozygous SNP (See Supplementary Figure S1). On the other hand Sanger sequencing of PCR-amplicons containing four putative heterozygous SNPs for *M. roreri* isolate C13 showed that there was no actual heterozygosity (See Supplementary Figure S2). Beside SNP 058_2_8046, the other three SNPs can be explained as misalignment between two or more paralogous genes. Though the SNP 485_1_1286 flanking region showed no paralogous genes during the initial BLASTn search of the genome, Sanger sequencing indicated the presence of two paralogous genes. As we continuously extended the sequence reads out from SNP 485_1_1286 we reached a point where there was no sequence homology and the SNP was invalidated (**Figure 2**). Therefore we selected 288 homozygous SNPs for validation by genotyping a test panel of 172 *M. roreri* isolates from the most important FPR affected areas of South and Central America. The overview of the SNP mining strategy from *M. roreri* RNASeq libraries shows the stringency involved at the various levels (**Figure 1**).

### Frequency of SNP Markers and Descriptive Statistics

Out of the chosen 288 SNP markers, 232 primer sets were successful in specific targeted amplification. The failure of the remaining 56 primer sets was likely due to the sequence complexity or the presence of polymorphisms within the flanking sequences. Though all the SNPs tested here were homozygous based on the *in silico* variant calling, Fluidigm genotyping analysis software called 69 SNPs as heterozygous. To validate the heterozygous SNP calls by the Fluidigm system, two such putative SNPs were tested from three *M. roreri* isolates using Sanger sequencing. Sanger sequencing invalidated the presence of heterozygosity (See Supplementary Figure S3). We further discarded the SNPs that were monomorphic or didn’t show consistent allelic intensity between two replicating experiments. A total of 88 polymorphic SNPs were retained for further analysis (See Supplementary Excel files S1 and S4). These 88 SNPs were reliably scored across the validation panel and thus, were considered true SNPs. The minor allele frequency of these SNPs ranged from 0.01 to 0.483 with an average of 0.218. There was no heterozygosity observed among any of the 88 SNP markers except in a very few instances which we attributed to the Fluidigm genotyping analysis software error, which was later established by Sanger sequencing. All these 88 SNPs can be repeated in genotyping to obtain fully identifiable genotypes.

### Genetic Relationship among *M. roreri* Isolates

**FIGURE 3:**
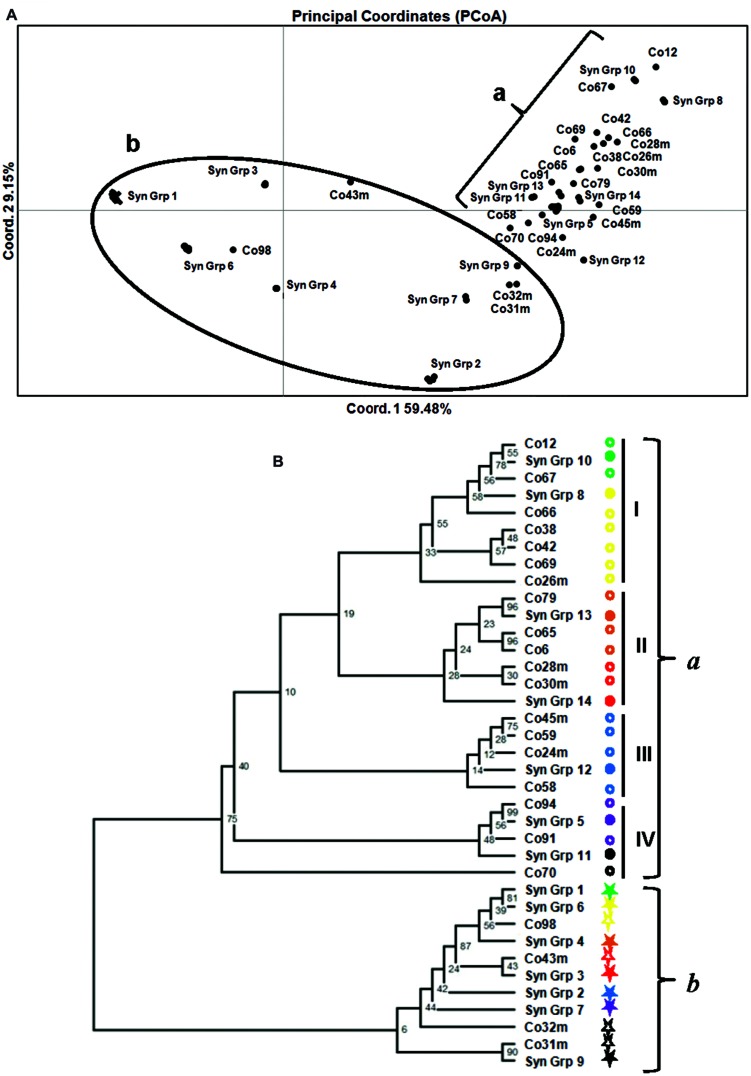
**Genetic relationships based on 88 SNP markers among 172 *M. roreri* isolates collected from frosty pod rot affected areas of South and Central America. (A)** Principal Coordinates Analysis based on the pairwise distance matrix. The plane of the first three main PCO axes accounted for 75.1% of total variation. First axis = 59.48% of total information, the second = 9.15%, and the third = 6.47%. **(B)** Cluster analysis and a consensus dendrogram were generated from the resulting distance matrix using the neighbor-joining algorithm (Saitou and Nei, 1987) and visualized using Figtree ver. 1.3.1 (Rambaut, 2009). Nei et al.’s (1983) genetic distance was calculated using microsatellite analyzer (Dieringer and Schlötterer, 2003) and 100 bootstrap replications were applied.

The genetic relationships among the 172 *M. roreri* isolates are presented in a PCoA plot (**Figure 3A**). Based on the PCoA results and aligning all the isolates according to the SNPs (See Supplementary Excel file S1), we can conclude that there are 37 groups of distinct SNP patterns and all the isolates within a group are synonymous (Syn Grp.). Among these 37 distinct genotypes (14 SynGrp. and 23 isolates with unique genotypes), 36 were present in Colombia followed by four in Ecuador (**Table 2**). All 86 Costa Rican isolates along with two Colombian and one Ecuadorian isolates were synonymous (Syn Grp. 1). While all the Bolivian and Peruvian isolates along with one Ecuadorian isolate formed another synonymous group (Syn Grp. 2). Treating each synonymous group as an individual isolate allows all of the 37 distinct genotypes to be grouped into two major clusters. One comprised most of the variant Colombian isolates along with two Ecuadorian isolates (E-06, and E-07), while the other cluster is mostly comprised of the synonymous groups derived from Costa Rican, Colombian, Venezuelan, Bolivian, and Ecuadorian isolates (**Figure 3A**).

**Table 2:** Geographic origin of 172 *Moniliophthora roreri* isolate.

Synonymous groups	Isolates and geographical origin
Syn Grp. 1	Co20m (Santander, Colombia), Co33 (Huila, Colombia), E32 (Guayas, Ecuador), and all 86 Costa Rican isolates.
Syn Grp. 2	B3, B1a, B4, B2b, B1b, B2a (La Paz, Bolivia), P-06, P-05 (Huánuco, Peru), and E21 (Manabí, Ecuador).
Syn Grp. 3	Co13m (Antioquia, Colombia), Co49, Co51 (Magdalena, Colombia), and V-08 (Zulia, Venezuela).
Syn Grp. 4	E-01m, E1b (Guayas, Ecuador), E24v, E27 (Esmeraldas, Ecuador), E-04, E44, E45 (Los Ríos, Ecuador), Co37m, and Co46 (Antioquia, Colombia).
Syn Grp. 5	Co76 (Valle del Cauca, Colombia), Co21m, Co52, Co23m, Co22, Co53, Co75 (Santander, Colombia), E-06, and E-07 (Los Ríos, Ecuador).
Syn Grp. 6	Co15 (Santander, Colombia), Co100, Co101, Co99 (Antioquia, Colombia), Co89, Co87 (Cesar, Colombia), and Co85 (Nariño, Colombia).
Syn Grp. 7	Co8 and Co44m (Antioquia, Colombia).
Syn Grp. 8	Co19m (Norte de Santander, Colombia), Co40, Co47, and Co47m (Antioquia, Colombia).
Syn Grp. 9	Co34, Co36m and Co63 (Huila, Colombia).
Syn Grp. 10	Co71 (Santander, Colombia) and Co77 (Valle del Cauca, Colombia).
Syn Grp. 11	Co29, Co80, Co81 (Santander, Colombia), and Co83 (Nariño, Colombia).
Syn Grp. 12	Co56, Co86 (Arauca, Colombia), and Co54 (Meta, Colombia).
Syn Grp. 13	Co82 (Santander, Colombia) and Co84 (Nariño, Colombia).
Syn Grp. 14	Co74 and Co25m (Santander, Colombia).
Isolates with unique genotype	Co6, Co24m, Co26m, Co28m, Co30m, Co70, Co79, Co91, Co94 (Santander, Colombia), Co38, Co42, Co43m, Co45m, Co98 (Antioquia, Colombia), Co31m, Co32m (Huila, Colombia), Co58, Co59 (Huila, Cundinamarca), Co65, Co66 (Norte de Santander, Colombia), Co67 (Risaralda, Colombia), Co69 (Tolima, Colombia), and Co12 (Caldas, Colombia).

*See Supplementary Excel file S1 for further details.*

**FIGURE 4:**
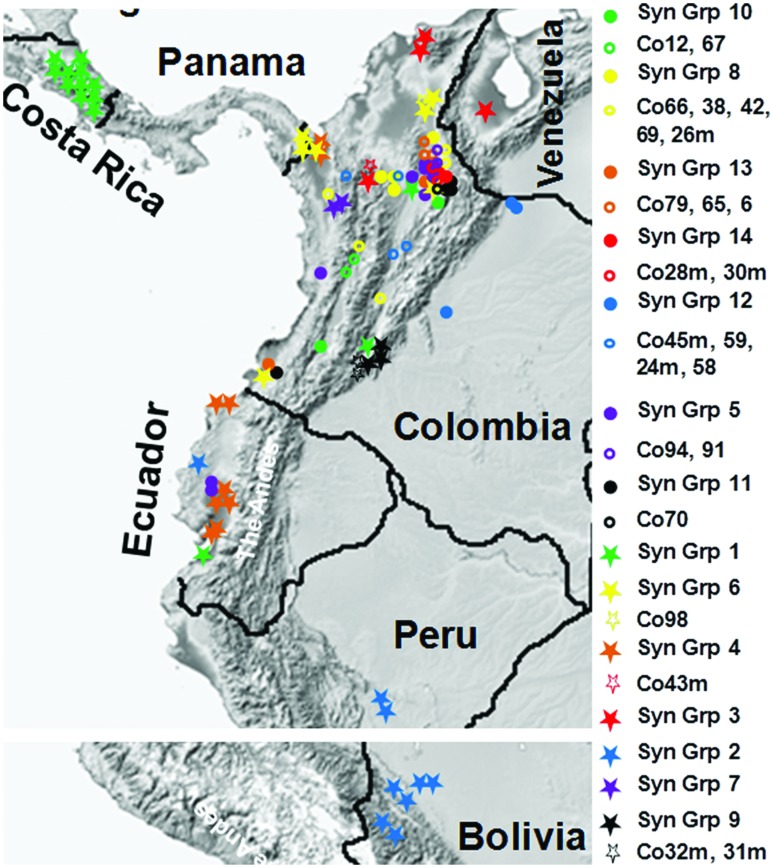
**Geographical distributions of *M. roreri* isolates collected from frosty pod rot affected areas of South and Central America and their phylogenetic relation.** Isolates were collected from infected cacao pods from 1999 to 2013. Approximate geographical locations were indicated using Google map application software. See **Figure 3** for phylogenetic relationships based on the color code and **Table 2** for synonymous group codes and isolates with unique genotypes.

A dendrogram was generated based on the SNP profiles for the synonymous groups and isolates with unique genotypes. This analysis increased the resolution and the degree of statistical support for the groups identified, with two discrete phylogenetic groups of isolates (*a* and *b*) supported by bootstrap values >50%, and another four phylogenetic sub-groups that were poorly supported, which were derived from Colombia isolates (**Figure 3B**). Approximate geographical locations for all of the 172 isolates are shown in **Figure 4**.

## Discussion

Due to the insufficient throughput and data standardization issues over time and between research facilities, the existing molecular marker-based genotyping technology for *M. roreri*, such as AFLP and SSR markers, is of limited use and a more robust and quicker analytical marker system is needed. Although whole genome sequencing may have generated more SNPs, the present study demonstrates the usefulness of next-generation sequencing (NGS) of RNA as a method of SNP detection using expressed genes of *M. roreri.* The developed SNP panel has already proven to be a powerful tool for studying the dispersion, evolution and epidemiology of this pathosystem, and will have a major impact on future studies designed to understand the interplay between pathogen genetic diversity and host resistance/tolerance.

The *M. roreri* isolates included in the RNASeq analysis were selected to represent all five of the genetic groups previously reported by Phillips-Mora et al. (2007a). To have a similar transcript pool and avoid complex fractions of the total transcriptomes, all of the isolates were cultured in a simple media (PDB) prior to RNA isolation. BLASTn searches of the SNP-flanking sequence for the 88 confirmed SNPs also didn’t show any bias toward any particular type of coding sequence (See Supplementary Excel file S4). It was interesting to see that 25% of the SNP containing transcripts had no homologies within the predicted CDS of the recently published *M. roreri* genome (Meinhardt et al., 2014). These new CDS were visible as viable transcripts based on sequence alignments implying that *M. roreri* has many more genes than previously thought.

The SNP discovery process for *M. roreri* was more difficult than anticipated. Only 88 of the putative SNPs identified by RNASeq analysis and selected for verification produced usable SNP markers. Though the process is considered to be robust, SNP discovery though NGS attempts have showed highly variable frequency of SNP validation, ranging from 48% in rainbow trout (Sánchez et al., 2009), 70% in the coral (Meyer et al., 2009), 83% in Eucalyptus (Novaes et al., 2008), and 85% in maize (Barbazuk et al., 2007). Moreover, the limited genetic variability observed among the *M. roreri* isolates studied herein may be one of the reasons for the restricted SNP validation. In general, the likely causes of erroneous SNP selection include sequencing errors, sample contamination from other species, biased sample pooling, and sequencing library construction (Tollenaere et al., 2012), and, as we have demonstrated, alignment of paralogs (Garvin et al., 2010). Because the SNP identification process was based on a comparison with the reference genome sequence MCA2977, it is logical, as observed, to expect very few homozygous SNPs with this isolate. But unlike other isolates, a high heterozygous to homozygous ratio (7.5) was obtained for *M. roreri* isolate MCA2977, which indicates the *in silico* heterozygosity might be a result of sequencing errors or more likely due to paralogous gene alignments as shown. Moreover *M. roreri* is thought to be sexually propagated through meiosis (Evans et al., 2002) in which case it should show both the homozygous and heterozygous SNP combinations for the same allele. But only seven such combinations were found among the 7,862 putative SNPs (See Supplementary Excel file S2). This further indicates that the heterozygosity we observed in the *in silico* results is not due to recombination but sequencing errors. In this case, we were able to verify alignment errors due to misalignments of reads between closely related genes and gene families. Indeed, when we visualized the SNPs using the bioinformatics suite SeqMan Pro of DNASTAR, a majority of the heterozygous SNPs resulted from misalignment of truncated reads (<50 bp) and/or were located within regions presenting additional flanking SNPs (data not shown). A BLASTn search against the *M. roreri* genome has further confirmed the postulation that the majority of these heterozygous SNPs are actually associated with misalignment between paralogous sequences instead of unique genes. Moreover, Sanger sequencing of partial genomic DNA fragments associated with specific SNPs has validated homozygous SNPs which vary between isolates but not heterozygous SNPs. Therefore we adopted a very stringent approach for the final SNP calling (**Figure 1**).

The present method of nanofluidic array of SNP markers can handle a large amount of samples in a short period of time and is highly robust and repeatable. The STA protocol (Wang et al., 2009) used in the study efficiently dealt with the quality or quantity of DNA extracted from the *M. roreri* isolates by performing a multiplex PCR reaction before genotyping, using primers for all loci of interest, but without targeting the specific alleles, thus proportionally increasing the copies of these loci. Results from the repeatedly genotyped isolates showed 100% concordance of allelic intensity for the 88 selected SNPs, and suggests that the nanofluidic system is a reliable platform for generating *M. roreri* isolate genotypes with high accuracy. In the few instances where the Fluidigm genotyping analysis software erroneously called heterozygous SNPs, it was observed that the allelic intensity of the non-template controls (NTC) for those SNPs were comparatively high. Therefore it is necessary to stringently check the NTC values for any SNP panel using the Fluidigm system.

If *M. roreri* reproduces sexually with any frequency as predicted previously by Evans et al. (2002) and were heterothallic, we should have seen heterozygosity among the 88 homozygous SNP markers tested here within the large population from a close geographical area like Colombia. The absence of heterozygosity for 88 SNP markers within Colombia suggests that there is no recombination occurring among the *M. roreri* isolates and would seem to make it highly unlikely that *M. roreri* reproduces by sexual recombination with any significant frequency. Phillips-Mora et al. (2007a) also observed very low recombination in *M. roreri* population analysis using AFLPs and ISSRs suggesting *M. roreri* reproduction is clonal. The present study confirms in unambiguous terms that *M. roreri* reproduction is clonal but does not rule out meiosis as a common occurrence in *M. roreri*.

During the biotrophic phase of *M. roreri* infection, the fruit develops malformations and then progresses to the necrotrophic phase where rot occurs, followed by sporulation on the pod surface (Evans et al., 2002; Evans, 2007). The spores are thought to be the product of meiosis without a basidiocarp (Evans et al., 2002). The mycelial mass that populates the pod during the biotrophic phase following initial infection is reported to be in a haploid state, while the formation of dikaryotic mycelia is thought to coincide with the necrotrophic phase of the disease and leads to sporulation and completion of the life cycle (Evans et al., 2002). The trigger for the shift from the biotrophic to the necrotrophic phase is unknown so far. Possible triggers include developmental signals from the host or quorum sensing by the fungus (Newton et al., 2010). It is also established that single spore generated cultures which are dikaryotic can produce infective spores capable of establishing the biotrophic phase. It is in these spores that Evans et al. (2002) found evidence of a highly modified form of meiosis. An intriguing possibility is that *M. roreri* reproduces by meiosis leading to a haploid biotrophic mycelium which has nuclei that auto duplicate thus forming the dikaryotic necrotrophic mycelia. This process would insure homozygosity is maintained for every reproductive generation under most circumstances.

The two Costa Rican isolates from late 1970s and early 1980s (ATCC42952 and ATCC64239) showed 100% sequence similarities with all the isolates collected in the country between 1999 and 2013, while the same genotype (Co20m, Co33, and E32) was also isolated from infected cacao pods in Colombia and Ecuador during 1999 and 2004. It clearly suggests that these particular *M. roreri* isolates were able to propagate clonally for more than three decades without recombination. During the movement of *M. roreri* into Central America apparently only one genotype was able to pass through, resulting in an obvious bottle neck. Similarly, it appears a single isolate crossed the Andes into Eastern Ecuador and then moved south toward Peru and Bolivia (**Figure 4**). Analysis of additional isolates should confirm this observation, although with time additional isolates may be introduced into these areas.

Whether a fungus reproduces clonally or sexually depends on various factors relating to its biology and distribution in space and time (Taylor et al., 1999). In the *M. roreri* sister taxon *M. perniciosa*, the presence of *A* and *B* mating type genes, normally associated with tetrapolar species, was used to demonstrate the evolution of self-compatibility in the presence of such genes (Kües and Navarro-González, 2010). Looking into the *M. roreri* genome, the presence of homeodomain transcription factors HD1 (XP_007858882) and HD2 (XP_007858883) along with nine potential pheromone receptors (XP_007851881, XP_007859042, XP_007849207, XP_007853337, XP_007854546, XP_007854545, XP_007854550, XP_007849212, and XP_007849206) indicates that it may also possess a tetrapolar mating system similar to *M. perniciosa*. Even though primarily homothallic Agaricomycetes have no obvious need for such mating type genes, they are widely present in homothallic species (Martinez et al., 2004; Kües and Navarro-González, 2010). In the case of *M. roreri* the fungus appears to have specific mechanisms to maintain a homokaryotic state. How and why this fungal pathogen is capable of functioning in a clonal state has yet to be determined but a series of heterokaryon incompatibility (het) proteins are up-regulated during the necrotrophic phase prior to spore formation which could be responsible for the homokaryotic nuclear state. These up-regulated proteins are het-c, het-e, nacht, and WD40 domain proteins and NWD2 proteins (Meinhardt et al., 2014), all of which are integral components of the somatic self/non-self recognition system that preserves genetic identity and prevents heterokaryon formation between unlike individuals through programmed cell death (Van der Nest et al., 2014). Though the present finding confirms reproduction is primarily through homothallism in *M. roreri* isolates causing disease on cacao, the possibility of heterothallism for some isolates within this species remains. Further studies in terms of nuclear contents of the spores and germ tubes during infection, biotrophic mycelia and necrotrophic mycelia are needed. The SNPs identified here in can be used for determining any possible recombination between divergent isolates in induced mixed infection studies.

The limited nature of resistance/tolerance against this fungus in the cacao germplasm (Phillips-Mora et al., 2005; Phillips-Mora, 2010) and the observed increases in disease losses in some tolerant clones in Costa Rica (Phillips-Mora et al., 2012; Bailey et al., 2014) raises the interesting question as to how a pathogen with low genetic diversity and limited genetic recombination potential can maintain such a highly virulent state. The answer likely lies in the unique origins of *M. roreri*, possibly as an endophyte, and its resulting genetic makeup (Meinhardt et al., 2014) combined with products of horizontal gene transfer (Tiburcio et al., 2010) and possibly viruses or retro elements acquired during evolution (Evans et al., 2013). It has also been suggested that the induction of *M. roreri* stress response genes during infection of tolerant clones may enable the fungus in overcoming cacao tolerance mechanisms (Bailey et al., 2014).

As the occurrence and distribution of genetically diverse groups in an area is the basis of designating centers of diversity and origin for the species, the present study suggest that Colombia is the likely center of origin for *M. roreri*, contradicting the previous hypothesis of Ecuador being the origin (Rorer, 1918; Briton-Jones, 1934; Erneholm, 1948). Phillips-Mora et al. (2007a) has also predicted the central/north-eastern Colombia as the origin. Based on the present SNP study, the upper Magdalena Valley, which contains 16 distinct genotypes out of which 10 are limited to this region showed the highest levels of genetic diversity within Colombia (**Figure 4**) and is most likely the center of origin of *M. roreri*. This was the area where FPR has been recorded on wild *Theobroma* by Baker et al. (1953), Cuatrecasas (1964) later identified it as *T. gileri*. Moreover, the presence of ancient populations of wild *Theobroma* and *Herrania* species known to be susceptible to *M. roreri* in this region of the Magdalena Valley of Colombia (Galán Gómez, 1947) further support the conclusion that this area is the center of origin of *M. roreri*. Based on the early reports from Ecuador and Colombia (Rorer, 1918; Baker et al., 1953), it seems *M. roreri* was endemic on wild *Theobroma* species of north-west Ecuador and western Colombia forests and that the cacao pathogen strain evolved in the latter region following a host jump, whilst the Ecuadorian *M. roreri* strains (var. *gileri*) never escaped from the sub-montane forests, and is non-pathogenic to cacao. It is probable that cacao was originally brought from its center of origin in the Upper Amazon and has been cultivated by the indigenous peoples of Magdalena Valley, Colombia for several thousands of years before it moved to other regions of Colombia (Bartley, 2005), and thus the crop has mingled with local *Theobroma* and *Herrania* species. This would have provided ample time for *M. roreri* to have jumped host and then evolved on cacao. Alternatively *M. roreri* might have evolved with a high degree of diversity within the wild *Theobroma* and *Herrania* species of Colombia and jumped multiple times to cacao after the commercial plantations started. Commercial cacao plantation of Colombia would have been at risk from the pathogen in the 19th century, hence the historical and catastrophic disease outbreaks reported in the 1850s with symptoms that resemble those of FPR (Ancízar, 1853; Baker et al., 1953; Phillips-Mora et al., 2007a). It also seems likely that the switch from the indigenous Nacional variety of western Ecuador to the more productive Forastero during the latter part of the 19th century (Rorer, 1918) led to the accidental importation of *M. roreri* from Colombia with disastrous consequences for the cacao industry and to the gradual abandonment of large-scale production of cacao in Ecuador (Evans, 1986). The previous suggestion of natural dispersal of *M. roreri* into Central America (Holliday, 1957) seems less likely and the movement of *M. roreri* into Central America along with the more recent occurrences in Peru and Bolivia probably resulted from a single introduction by humans as suggested by Phillips-Mora et al. (2007a).

Unlike the previously reported five genetic groups (Phillips-Mora et al., 2007a), the distance based PCoA and dendrogram analysis carried out here, grouped the 172 isolates into two major phylogenetic groups, one being highly dispersed both in terms of genetic variation and geographical distance (Group *b*) while the other group (Group *a*) is mainly confined to Colombia, and has significant genetic diversity resulting in many unique isolates (Group *a*; **Figure 3**). The low bootstrap support for the four phylogenetic Sub-groups within Group *a* was due to small number of SNPs involved in those differentiations. As each SNP is a unique event that can be tracked with certainty, the genetic differentiations here in is of high confidence. The phylogenetic Sub-group I is centered around the upper Magdalena Valley and crossed the Central Andes through the northern side and then moved south along the Cauca Valley. Similarly, Sub-groups II and IV moved west from upper Magdalena Valley and crossed the Western Andes and the moved south along the coast reaching Ecuador (**Figures 3** and **4**). On the other hand phylogenetic Sub-group III was able to cross the Eastern Andes. Similarly, isolates of phylogenetic Group *b* were isolated from the upper Magdalena Valley. They moved North and West from the upper Magdalena Valley, while a few isolates were able to move south along the coast (**Figure 4**). In a previous finding by Phillips-Mora et al. (2007a), 12 Colombian isolates had been grouped into two genetic groups confined to central Colombia and Santander regions; the present study shows a more diverse picture of the pathogen in terms of distribution within and dispersal from the Santander regions. Since we now know *M. roreri* maintains a clonal state, we expect to see less indication of genetic drift associated with the movement from the pathogens center of origin. Instead, this study suggested dispersal of *M. roreri* clones from the upper Magdalena Valley over time and the subsequent localized genetic drift associated with these unique isolates and events.

It may be that natural dispersal started out on the wild *Theobroma* and *Herrania* hosts (Holliday, 1957; Evans, 1981) and then jumped to commercial cacao. Dispersal by the early civilizations and settlers is another probability especially if we consider the huge physical barrier created by the Andes within Colombia. The question remains, why isolates those have been successful in disseminating, show greater genetic diversity and essentially appear as out groups compared to the isolates of limited distribution from within Colombia and Ecuador. Fungal spores are the single most important part of the *M. roreri* life cycle when it comes to dispersion in time and space. Though comparison of spore shape and size of these two groups suggest no obvious difference (See Supplementary Figure S4), they might differ in composition of the compatible solutes and other protective proteins inside the spores which are influenced by the environmental conditions during their formation (Wyatt et al., 2013). For a clearer picture of genetic or geographic pattern of dispersion within Colombia, more isolates, especially from the regions other than upper Magdalena Valley, should be included in future studies. For population studies involving countries other than Colombia we need to incorporate more SNPs derived from comparing isolates collected in those countries due to their limited diversity and the clonal nature of SNP dispersal.

## Conclusion

The current set of SNP markers developed for *M. roreri* can be widely used for population genetics and evolutionary studies of the pathogen and would be a great asset for breeding cacao tolerant against to FPR. The fact that *M. roreri* propagation is clonal or homothallic and the generation of new recombinants through sexual propagation is a comparatively rare event, if it occurs at all, has a huge implication on the breeding and disease management strategy against FPR. The present study also gives credence to the need for strict quarantine even in areas where FPR is endemic to stop the introduction of new *M. roreri* genotypes which might make the disease management more complicated. Being the probable center of origin and representing almost half of the entire genetic diversity, the upper Magdalena Valley of Colombia becomes the most important area in terms of FPR research. The establishment of breeding and screening programs in this area would assure new cacao germplasm was selected for tolerance against the widest *M. roreri* genetic base.

## Conflict of Interest Statement

The authors declare that the research was conducted in the absence of any commercial or financial relationships that could be construed as a potential conflict of interest.
