# Supplementary material for: Combination of RNAseq and SNP nanofluidic array reveals the center of genetic diversity of cacao pathogen Moniliophthora roreri in the upper Magdalena Valley of Colombia and its clonality
Source: Front Microbiol. 2015 Aug 27;6:850. doi: 10.3389/fmicb.2015.00850 (PMC4550789; doi:10.3389/fmicb.2015.00850)
Supplement: Supplementary file 1 [file Data_Sheet_1.ZIP › All supplementary materials/Supplementary Material.docx]

***Supplementary Material***

**Combination of RNAseq and SNP nanofluidic array reveals the center of genetic diversity of cacao pathogen *Moniliophthora roreri* in the upper Magdalena Valley of Colombia and its clonality.**

Shahin S. Ali^1^, Jonathan Shao^1^, Mary D. Strem^1^, Wilberth Phillips-Mora ^3^, Dapeng Zhang^1^, Lyndel Meinhardt^1^ and Bryan A. Bailey^1*^

^1^Sustainable Perennial Crops Laboratory, Plant Sciences Institute, USDA/ARS, Beltsville Agricultural Research Center-West, Beltsville, MD 20705, USA.

^2^Departamento de Agricultura y Agroforestería, Centro Agronómico Tropica de Investigación y Enseñanza (CATIE), 7170 Turrialba, Costa Rica.

*Corresponding author:

Bryan A. Bailey

USDA/ARS, Beltsville Agricultural Research Center.

Beltsville, MD 20705

Phone: 1-301-504-7985; Fax: 1-301-504-1998

E-mail: bryan.bailey@ars.usda.gov

**Supplementary Figures**

**Figure S1** Sanger sequencing of partial genomic DNA containing putative homozygous SNP marker (002_9_133533) as called by SAMtools mpileup and bcftools with default parameters from 3 different *Moniliophthora roreri* isolates (C7, C19 and P5). PCR-amplicons were cloned into pCR4-TOPO vector followed by transformation in to DH5-alpha *E. coli* and 9 to 11 clones from each amplicon were randomly selected to be sequenced from both ends using M13F and M13R primers. ClustalW2 (Larkin *et al.*, 2007) was used to compare the DNA sequences of each clone and the original sequences containing the SNP.

**Figure S2** Sanger sequencing of partial genomic DNA containing 4 putative heterozygous SNP markers (**A.** 058_2_8046, **B.** 485_1_1286, **C.** 075_6_22587 and **D.** 052_1_6589) as called by SAMtools mpileup and bcftools with default parameters from *Moniliophthora roreri* isolates C13. PCR-amplicons were cloned into pCR4-TOPO vector followed by transformation in to DH5-alpha *E. coli* and 9 to 11 clones from each amplicon were randomly selected to be sequenced from both ends using M13F and M13R primers. ClustalW2 (Larkin *et al.*, 2007) was used to compare the DNA sequences of each clone and the original sequences containing the SNP and a distance tree of 100 bootstrapped data sets was generated by using the Phylogeny.fr program (http://phylogeny.lirmm.fr) and the neighbor-joining method.

**Figure S3** Sanger sequencing of partial genomic DNA containing two putative heterozygous SNPs markers (**A.** 034_4_15170 and **B.** 061_2_74886) as shown by the Fluidigm genotyping analysis software from 3 different *Moniliophthora roreri* isolates. PCR-amplicons were cloned into pCR4-TOPO vector followed by transformation in to DH5-alpha *E. coli* and 9 to 11 clones from each amplicon were randomly selected to be sequenced from both ends using M13F and M13R primers. ClustalW2 (Larkin *et al.*, 2007) was used to compare the DNA sequences of each clone and the original sequences containing the SNP.

**Figure S4** *Moniliophthora roreri* spore and colony morphology of the two phylogenetic groups based on SNP marker analysis. *M. roreri* isolates from the geographically dispersed phylogenetic group are **A.** ATCC42952 (Syn Grp. 1), **B.** B3 (Syn Grp. 1), **C.** Co15 (Syn Grp. 6) and **D.** Co46 (Syn Grp. 4); while isolates from phylogenetic group which is confined to Colombia are **E.** Co45m, **F.** Co58, **G.** Co67 and **H.** Co71 (Syn Grp. 10).

**Supplementary Tables**

**Table S1** Primers used for PCR amplification and cloning of SNP flanking regions.

| **SNP id** | **Forward primer** | **Reverse primer** | ***M. roreri* Contig no.** |
| --- | --- | --- | --- |
| 027_2_223188 | AGCAGCTACCTGGCAATCAC | CATCCAGAGAACCTCGCAGT | sctg_0027_0002 |
| 034_4_15170 | TACCCCAGACCGTGATCTTC | TCTTTCCACCCCTGCATATC | sctg_0034_0004 |
| 061_2_74886 | ATGGCGACGTAAAGAGCATT | ACCGCCAATAGCATCATCTT | sctg_0061_0002 |
| 058_2_8046 | GATCGACCTTGCTTCGTTTA | AGTAGCACCCGTCAACCAAC | sctg_0058_0002 |
| 485_1_1286 (P1) | GATCATGAGTTTGGGCGATT | AATGAGCTGTGCATCTGTGC | sctg_0485_0001 |
| 075_6_22587 | CCAGGCCTTCCTTTATTCCT | GCGTGTGTTTATAGTCCTTTCG | sctg_0075_0006 |
| 052_1_6589 | GATTTGGGAGAGGTGCTTCA | CTCGGCCCAAGCTGATATT | sctg_0052_0001 |
| 485_1_1286 (P2) | AGCTCAAAACATGCGTGATG | ACAAACCAATTTTGCCCTTG | sctg_0485_0001 |
| 485_1_1286 (P3) | ATCCAAGTGTCCGTCTCACC | GCAATGAGCTGTGCATCTGT | sctg_0485_0001 |
| 485_1_1286 (P4) | TGTCGCTCATCTGCTTCATC | CGTCTTGCGTCACAGGAATA | sctg_0485_0001 |

Primers were designed using the Primer3 software (version 0.4.0; http://frodo.wi.mit.edu/primer3/).
